# Poly-L-ornithine promotes preferred differentiation of neural stem/progenitor cells via ERK signalling pathway

**DOI:** 10.1038/srep15535

**Published:** 2015-10-27

**Authors:** Hongfei Ge, Liang Tan, Pengfei Wu, Yi Yin, Xin Liu, Hui Meng, Gaoyu Cui, Nan Wu, Jiangkai Lin, Rong Hu, Hua Feng

**Affiliations:** 1 Department of Neurosurgery and Key Laboratory of Neurotrauma, Southwest Hospital, Third Military Medical University, Chongqing 400038, China

## Abstract

Neural stem/progenitor cells (NSPCs) replacement therapies are the most attractive strategies to restore an injured brain. Key challenges of such therapies are enriching NSPCs and directing them differentiation into specific neural cell types. Here, three biomaterial substrates Poly-L-ornithine (PO), Poly-L-lysine (PLL) and fibronectin (FN) were investigated for their effects on proliferation and differentiation of rat NSPCs, and the underlying mechanisms were also explored. The results showed PO significantly increased NSPCs proliferation and induced preferred differentiation, compared with PLL and FN. Checking protein markers of several neural cell subtypes, it is showed PO significantly induced NSPCs expressing Doublecortin (DCX) and Olig2, one for neuroblasts and young neurons and the other for young oligodendrocytes. It is suggested the ERK signaling pathway was involving in this process because an ERK antagonist U0126 could inhibit PO’s effects mentioned above, as well as an ERK pathway agonist Ceramide C6 could enhance them. Given that both neurons and oligodendrocytes are the most vulnerable cells in many neurological diseases, PO-induced preferred differentiation into neurons and oligodendrocytes is a potential paradigm for NSPCs-based therapies.

Neural stem/progenitor cells (NSPCs), known as their characteristics of self-renewal, undifferentiated features and capacity to give birth to all neural lineage cell types^1^,^2^,^3^, especially neuronal subtypes^4^,^5^,^6^,^7^,^8^,^9^,^10^,^11^, have aroused much attention because of their potential to ameliorate various neurological diseases and injuries, meanwhile, provide a suitable cell source for transplantation therapies^12^. Currently, there are two culture systems to obtain NSPCs known as neurosphere culture system^13^,^14^ and adherent culture system coated with different substrates^15^,^16^,^17^,^18^. Especially, different substrates employed in adherent culture system, which could mimic the physiological microenvironment for culturing and expanding stem cells, have attracted great interests to date.

Although many substrates have been tried to fulfill above purpose including poly-L-ornithine (PO)^19^, Poly-L-Lysine (PLL)^20^, Laminin^21^,^22^,^23^, and Fibronectin (FN)^19^, their effects on proliferation and differentiation of NSPCs still remain elusive and which one is better for enriching NSPCs and directing their differentiation into preferred neural subtypes needs to be fully explored. PLL alone or combined with FN or laminin is widely used for the attachment of NSPCs to explore their morphology, proliferation, migration and differentiation^24^. While, some studies suggest that PLL could enhance the likelihood of host inflammatory responses^25^. Recent studies have shown that PO is less immunogenic than PLL and possesses some other advantages^26^,^27^. FN, which is an extracellular glycoprotein that binds both cell integrins and other ECM molecules, plays a pivotal role in cell adhesion, survival and differentiation^28^. However, Sun, T. *et al.* report that NSPCs on FN-coated dish, lose their proliferation potential after P6^29^. As a result, it is necessary to explore the outcomes caused by different substrates on the biological behaviors of NSPCs.

In this present study, three substrates (PO, PLL and FN) were tested for their ability to promote proliferation and preferred differentiation of NSPCs. Meanwhile, the likely underlying mechanism(s) was explored. The aim of this study is to investigate their different effects on the proliferation and differentiation of NSPCs and therefore look for a more suitable biomaterial candidate to mimic the physiological microenvironment for expanding NSPCs and directing their preferred differentiation. At the same time, try to elucidate the possible underlying mechanism(s), which might provide a favorable cell replacement strategy for basic and clinical research associated with various neurological diseases and injuries.

## Results

### Culture and immunofluorescence identification for NSPCs

**Figure 1:**
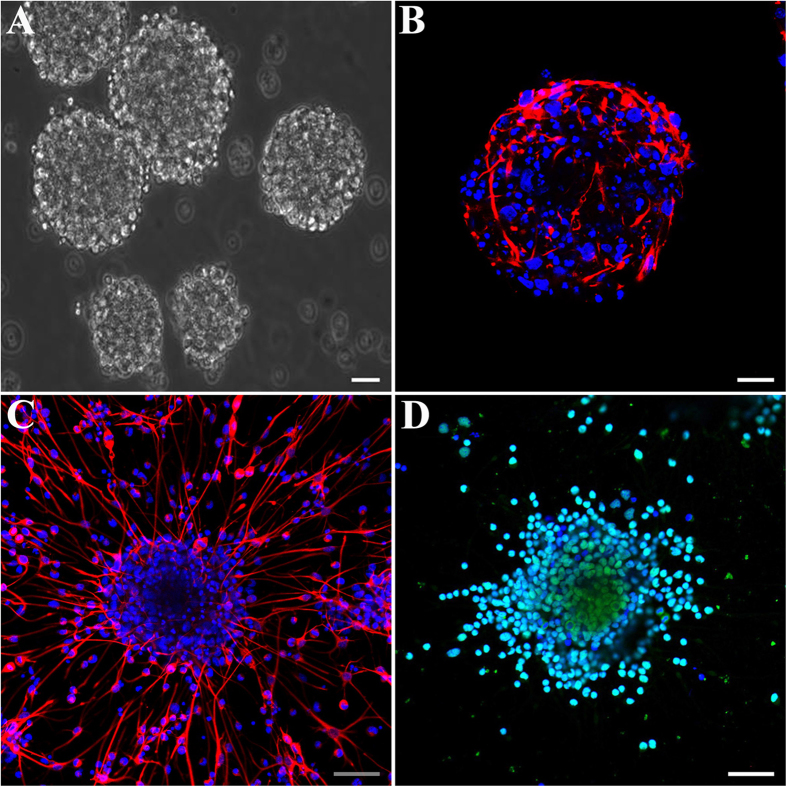
Cell morphology and molecular marker expressions of rat NSPCs. (A) The suspended growth of neurospheres was notably observed. (**B**) The immunostaining of the Nestin expression (red) of the NSPCs in neurosphere before seeded on substrates. (**C**) The immunostaining of the Nestin expression (red) of the NSPCs after seeded on PO. (**D**) The immunostaining of the Sox2 expression (green) of the NSPCs after seeded on PO. Cell nuclei was stained with DAPI in blue. Scale bar: 20 μm.

For preparation of NSPCs, fresh tissues were dissected from neocortical tissues from E14.5 Sprague–Dawley rats. The suspended growth of neurospheres was notably observed after 3 days cultured in the neurosphere culture system (Fig. 1A). Meanwhile, cells expressing Nestin, a marker for NSPCs, reached to 50–60% of total cells in a neurosphere, which was consistent with the previous study^15^ (Fig. 1B). In the adherent culture system, NSPCs also expressed Nestin (Fig. 1C, red) and Sox2 (Fig. 1D, green), another marker for NSPCs, after seeded on PO, PLL or FN (data not shown).

### PO, PLL and PN showed no different effects on survival of NSPCs

**Figure 2:**
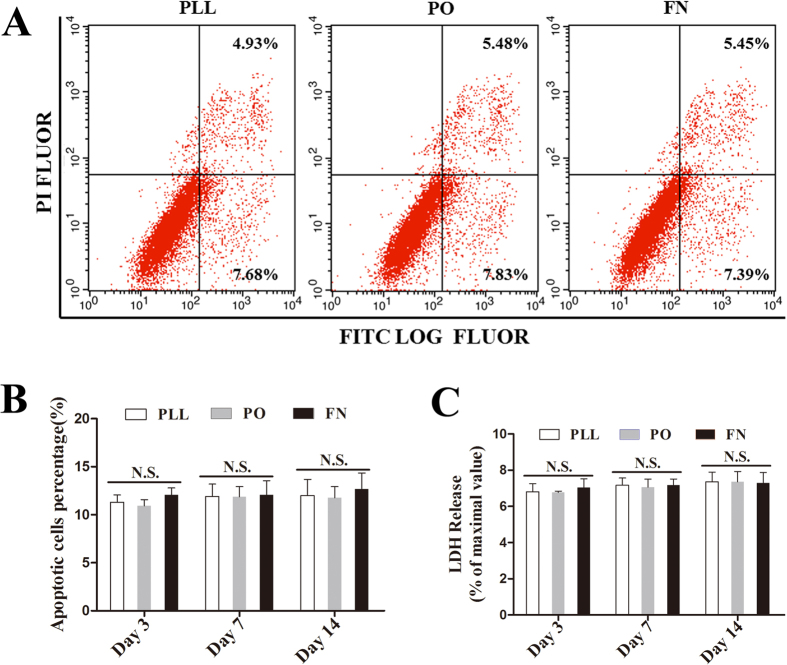
PO, PLL and PN showed no different effects on death/survival of NSPCs. (**A**) Typical illustrations showing death of NSPCs growing on PLL, PO and FN respectively, through flow cytometry on day 14 (n = 5). (**B**) Summarized graph showing the quantitative results from flow cytometry assay on day 3, 7 and 14 (n = 5). (**C**) Cell death of NSPCs determined by LDH assay on day 3, 7 and 14 growing on PLL, PO and FN respectively (n = 6). N.S. indicates no significant difference among PLL, PO and FN.

First, we evaluated the effects of PO, PLL and FN on the death/survival of NSPCs by using cell death/survival assays through flow cytometry. As shown in Fig. 2, though there was a tendency that there were more cell death with the prolonged culture time, no considerable difference was observed among PO, PLL and FN on day 3, 7 and 14 post cultured. The data suggested that these substrates have no different influence on the death/survival of NSPCs.

### PO significantly increased proliferation of NSPCs

**Figure 3:**
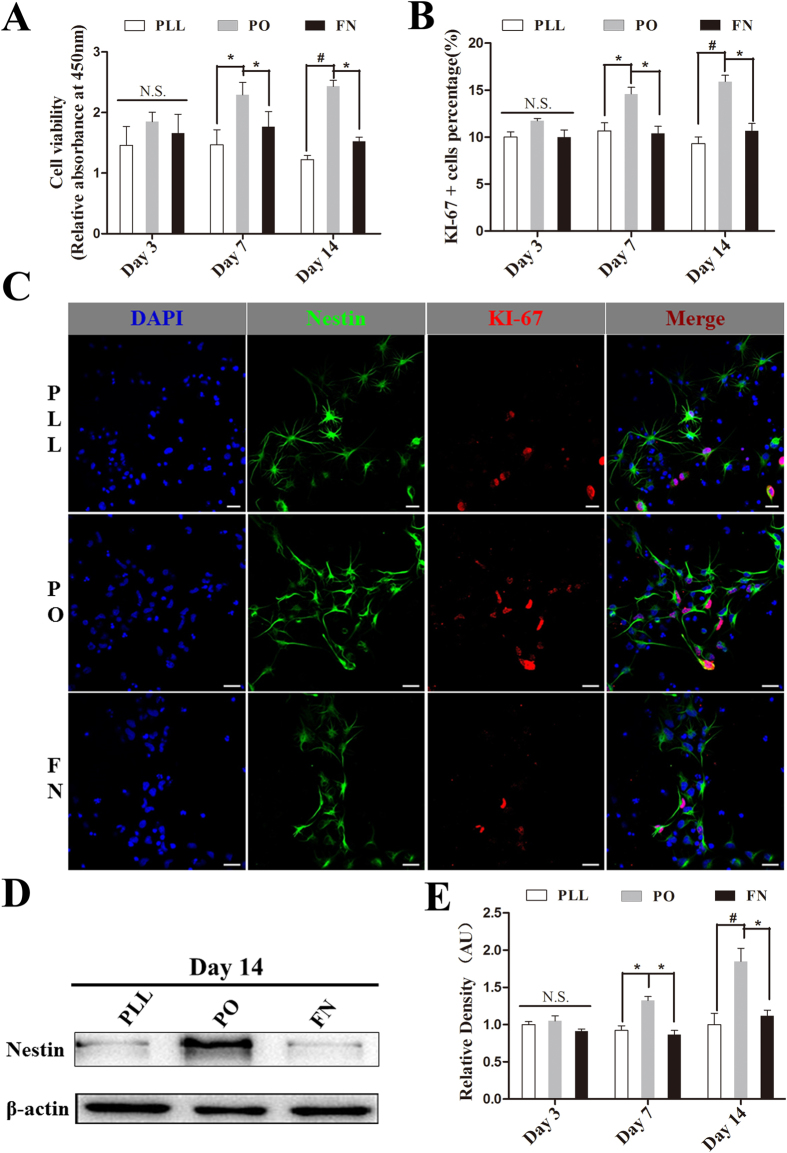
PO significantly increased proliferation of NSPCs. (**A**) Bar graph showing the proliferation of NSPCs growing on different substrates by using CCK8 assay on day 3, 7 and 14 (n = 6). *P < 0.05, ^#^P < 0.01, One-Way ANOVA followed by Tukey’s post hoc test. N.S. indicates no significant difference. (**B**) Bar graph showing the proliferation ability of NSPCs growing on different substrates by using Ki-67 assay on day 3, 7 and 14 (n = 3). *P < 0.05, ^#^P < 0.01, One-Way ANOVA followed by Tukey’s post hoc test. N.S. indicates no significant difference. (**C**) Immunostaining images showing the percentage of Ki-67 positive cells in NSPCs growing on PO was higher than that on PLL or FN. The NSPCs were stained with antibody for Ki-67 (red) and antibody for Nestin (green), cell nuclei was stained with DAPI in blue. Co-labeling of Ki-67 and Nestin suggests actively proliferating NSPCs. Scale bar: 20 μm. (**D**) Western blotting showing the level of Nestin expressed by NSPCs growing on different substrates on day 14. (**E**) Summarized graph showing the expression level of Nestin of NSPCs growing on PO was markedly higher than that on PLL or FN on day 7 and 14. Blotting bands were analyzed using the Image Lab™ software for relative density and normalized to β-actin controls. *P < 0.05, ^#^P < 0.01, One-Way ANOVA followed by Tukey’s post hoc test. N.S. indicates no significant difference.

Next, we investigated the effects of PO, PLL and FN on the proliferation of NSPCs. Here, NSPCs were cultured in the enrichment medium with 20 ng/ml bFGF and 20 ng/ml EGF according to the well-known standard method. First, CCK8 assay illustrated that the absorbance at 450nm was higher in NSPCs growing on PO on day 7 and 14 in comparison with PLL or FN (Fig. 3A), which implied that NSPCs growing on PO proliferated more vigorously than those on PLL or FN. Second, Ki-67 assay showed that the co-labeling percentage of Ki-67 and Nestin in NSPCs cultured on PO was significantly higher than that on PLL or FN on day 7 and 14 (Fig. 3B, C). Third, western blotting data showed that the expression level of Nestin of NSPCs growing on PO, which was preferentially expressed in NSPCs, was markedly higher than that on PLL or FN on day 7 and 14 (Fig. 3D, E). Together, these data indicated that PO has a better ability to promote the proliferation of NSPCs comparing with PLL or FN.

### PO induced preferred neuronal and oligodendrocytic differentiation of NSPCs

**Figure 4:**
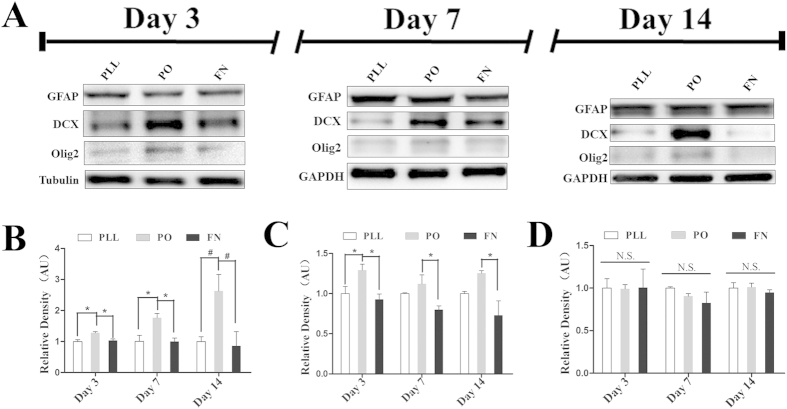
PO induced preferred neuronal and oligodendrocytic differentiation of NSPCs. (**A**) Western blotting bands showing three neural lineage differentiation of NSPCs by checking specific protein markers (DCX for young neurons, GFAP for astrocytes and Olig2 for oligodendrocytes) on day 3, 7 and 14. (**B–D**) Bar graphs summarizing quantitative results from (**A**) on day 3, 7 and 14, respectively. Bands were analyzed using the Image Lab™ software for relative density and normalized to Tubulin or GAPDH controls. *P < 0.05, ^#^P < 0.01, One-Way ANOVA followed by Tukey’s post hoc test. N.S. indicates no significant difference.

To investigate their effects on the differentiation of NSPCs, western blotting assays were performed. During this process, NSPCs were cultured in differentiation medium without 20 ng/ml bFGF and 20 ng/ml EGF. As shown in Fig. 4A, B, the expression of Doublecortin (DCX), which is preferentially expressed in neuroblasts and as the marker of young neurons, obviously increased in NSPCs growing on PO at all checking time points compared with PLL or FN. Their effects on glial cell fate, such as astrocyte or oligodendrocyte, were also examined. The expression level of glial fibrillary acidic protein (GFAP), which is widely used to identify astroglia in the brain, had no significant difference at all time points among different substrates (Fig. 4A, D). However, the expression of Olig2, expressed by young oligodendrocytes, significantly increased in NSPCs cultured on PO on day 7 and 14, compared to PLL or FN (Fig. 4A,C). These data indicated that NSPCs cultured on PO were more likely to give birth to young neurons and oligodendrocytes, representing a better candidate directing neuronal or oligodendrocytic differentiation of NSPCs.

### ERK activation involves in PO-induced preferred neuronal differentiation from NSPCs

**Figure 5:**
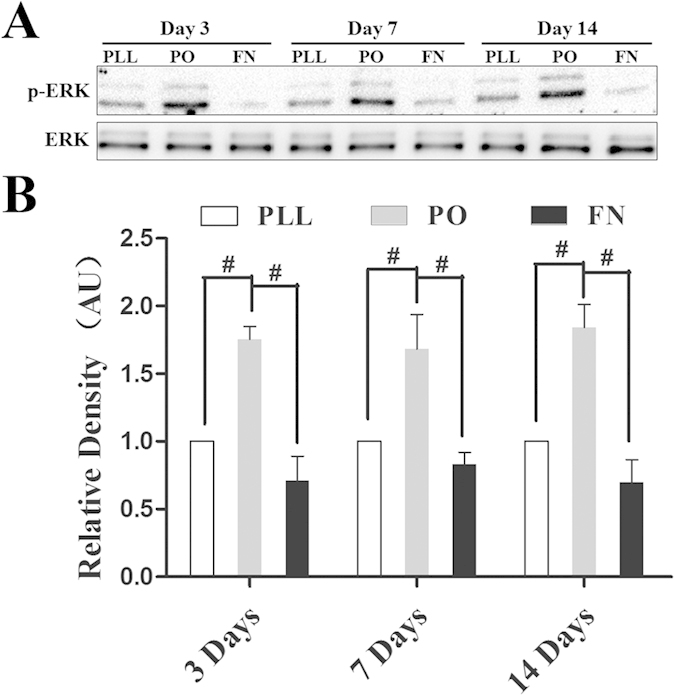
Activation of ERK was increased in NSPCs growing on PO. (**A**) Blotting bands showing p-ERK expression in NSPCs growing on PLL, PO and FN on day 3, 7 and 14. (**B**) Bar graphs summarizing quantitative results from (**A**). Bands were analyzed using the Image Lab™ software for relative density and normalized to total ERK controls. ^#^P < 0.01, One-Way ANOVA followed by Tukey’s post hoc test.

**Figure 6:**
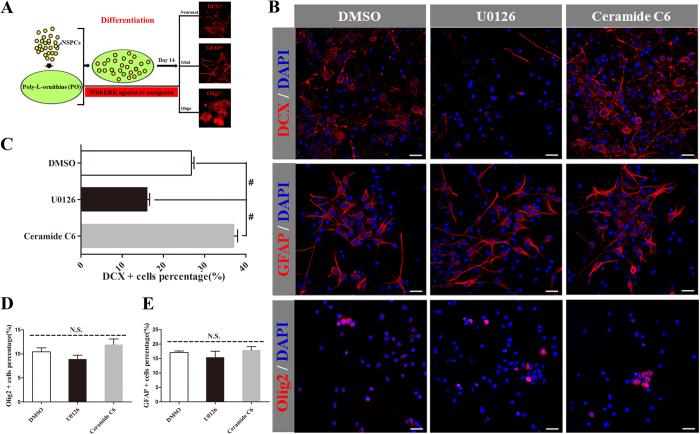
The crucial role of ERK in NSPCs differentiation into neuronal linage. (**A**) Illustration showing the experimental procedure. (**B**) Immunostaining images displaying the influence of ERK signalling on the differentiation of NSPCs growing on PO, PLL and FN into DCX^+^, GFAP^+^ and Olig2^+^ cells using ERK antagonist or agonist on day 14. Scale bar: 20 μm. (**C–E**) Bar graphs summarizing quantitative results from (**B**). ^#^P < 0.01, One-Way ANOVA followed by Tukey’s post hoc test. N.S. indicates no significance.

ERK has been reported to be implicated in the ECM-stimulated (including cell culture substrate) cell biological behaviors such as cell growth, proliferation and differentiation^30^. To unravel the potential mechanisms for preferential fate choice of NSPCs induced by PO, and whether ERK signaling pathway was involved in this process, the following tests were conducted. The activation of ERK was evaluated via measuring ERK phosphorylation (p-ERK) in western blot assay. As shown in Fig 5A, B, the expression of p-ERK in NSPCs cultured on PO was remarkably higher than that on PLL or FN at all checking time points. To provide direct evidence for the involvement of ERK in PO-induced preferred neuronal/oligodendrocytic differentiation, NSPCs cultured on PO were treated with a specific ERK antagonist U0126 (10 μM) or agonist Ceramide C6 (10 μM). Immunostaining data showed that antagonist significantly reduced the number of DCX^+^ cells (Fig. 6B, C) compared with the vehicle control. However, it had no obvious effect on number of GFAP^+^ cells (Fig. 6B, D) or Olig2^+^ cells (Fig. 6B, E). Instead, with the addition of 10 μM Ceramide C6 could enhance more differentiation of NSPCs growing on PO into young neurons obviously (Fig. 6B, C). However, there was no effect on the astroglial and oligodendrocytic differentiating capacity (Fig. 6B, D, E). Taken together, these data indicated that the activation of ERK signaling pathway might contribute to PO-induced preferred differentiation of NSPCs into neurons, but not oligodendrocytes.

## Discussion

The utility of neural stem/progenitor cells (NSPCs), with the ability of self-renewal and differentiation into functional neural cells^4^,^5^,^6^,^7^,^8^,^9^,^10^,^11^,^31^, holds promise for cell therapy-based treatment of many neurological disorders such as Parkinson’s disease^32^, traumatic brain injury^33^ and ischemic attack^34^,^35^,^36^. How to expand NSPCs and induce preferred differentiation into specific neural cell lineage is an attractive field to date. The adherent culture system with substrate(s) could mimic the physiological microenvironment for culturing and highly efficient expansion of NSPCs. Among the widely used substrates for culturing NSPCs such as PO, PLL and FN, this study, to our limited knowledge, for the first time demonstrates that PO promotes proliferation of NSPCs and preferential differentiation into neuronal and oligodendrocytic cell types, compared with PLL or FN. Given that both neurons and oligodendrocytes are the most vulnerable cells in many neurological diseases and injuries, PO-induced preferred differentiation into neurons and oligodendrocytes is a favorable strategy for NSPCs-based therapies.

During last decades, PO, PLL and FN are the most widely used culturing substrates for investigating NSPCs^24^. They can provide a good physical support for the attachment of NSPCs, therefore benefit survival of NSPCs in culture conditions. Few studies compared their effects on the proliferation and preferred differentiation of NSPCs. However, recent findings make comparative study necessary. For example, some evidences indicate that PLL is likely to enhance inflammatory responses^25^. Furthermore, it’s reported that adherent NSPCs on FN would lose their potentiality of proliferation after P6^29^. These evidence suggest that different biomaterials might have distinct effects on characteristics of NSPCs. In this study, we found PLL, FN, PO had no different effects on the survival of NSPCs, as reported previously^37^,^38^. Furthermore, we indeed found PO promoted the proliferation of NSPCs and preferential differentiation into neuronal and oligodendrocytic cells, compared to PLL or FN. In addition, previous studies have found that PO coating significantly increases the mechanical strength of the alginate microcapsule, which could provide a physical support for the attachment of NSPCs during cell-based therapy^24^ and results in lower shear stress to increase biocompatibility^39^. While, Flanagan LA *et al.* revealed that human NSPCs grown on PO for 5 days were less well spread^18^. The reason of this discrepancy between the two results might be the difference in coating procedure, cell density and difference in species. However, it provides a clue for the combination of PO and laminin for the culture of NSPCs and even for the improvement of biomaterials. Together, these data suggest PO hold the potential serving as a better biomaterial candidate than PLL and FN for enriching neurons and/or oligodendrocytes from NSPCs to treating neurological disorders in the future.

Although these conventional biomaterials are widely employed, the underlying mechanisms whereby NSPCs binding to substrates and undergoing proliferation and differentiation remain open. Usually, substrates in ECM offer outside-in signals for cells mediated by an array of integrins on the cell surface. Especially, β_1_-integrin receptor (such as α_5_β_1_, α_3_β_1_ and α_6_β_1_ integrin complexes^40^,^41^,^42^) has been shown to be crucial for NSPCs proliferation and neurogenesis^43^,^44^. However, the underlying mechanisms for how integrins transmit the signals across the cell membrane to cytoplasm remain elusive. Data in this research have shown the activation of ERK1/2 is required for PO-induced preferred neuronal differentiation from NSPCs. It has been reported that ERK1/2 is implicated in neuronal differentiation^30^,^45^,^46^. Therefore, it is needed to be further investigated how ERK1/2 acts with integrins to mediate PO signaling preferred neuronal differentiation. However, the PO-induced preferential differentiation into oligodendrocyte from NSPCs is not dependent on ERK1/2 activation, because the number of Olig2^+^ cells differentiated from NSPCs is not affected by the addition of ERK1/2 antagonist or agonist. This implies the mechanism whereby PO induced preferred oligodendrocytic differentiation is different from neuronal one. The mechanism(s) underlying PO-induced oligodendrocytic differentiation is not clear and needs to be determined in the future research.

In summary, our study has revealed pivotal insights into the activity of PO on NSPCs proliferation and differentiation, and might open new avenues for NSPCs-based therapies specifically promoting neuronal and oligodentrocytic differentiation in brain disorders.

## Methods

### Animal

This study was performed in accordance with the China’s animal welfare legislation for the care and use of animals and approved by the Third Military Medical University Chongqing, China. We did our best to minimize the number of animals and decrease their suffering. E14.5 Sprague-Dawley rats were sacrificed after anesthetized with pentobarbital (60 mg/kg intraperitoneally).

### Primary Neural Stem/Progenitor Cells Culture

Neocortical tissues obtained from E14.5 Sprague–Dawley rats were dipped in 0.25% Trypsin Protease (HyClone™, SV30031.01) for 30 minutes at 37 °C. Soybean trypsin inhibitor (2.8 mg/mL, Roche Diagnosis, Mannheim, Germany) was in addition to inhibit trypsin activity. After washing twice with DMEM/F12 medium(HyClone™, SH30023.01), the tissue samples were triturated using a fire-polished Pasteur pipette and passed through a 100 μm Nylon cell strainer (BD Faclcon, 352360) to harvest dissociated cell suspensions. Then, they were seeded at an initial cell density of 1 × 10^5^ cells/mL in DMEM/F12 medium supplemented with 20 ng/ml recombinant murine FGF-basic (PeproTech, 450–33), 20 ng/ml recombinant murine EGF(PeproTech, 315–09), and 2% B-27 supplement(Life Technologies, 17504–044) for 24 hours at 37 °C under humidified 5% CO2 conditions. NSPCs suspension were plated onto 35-mm different pre-coated dishes (18 hs) after washing twice with sterile cell culture grade water according to the manufacturer’s instructions respectively. The pre-coated substrates were as follows: 100 μg/ml Poly-L-lysine (sigma, P4832), 10 μg/ml poly-L-ornithine (Sigma, P4957) and 20 μg/ml Fibronectin(sigma, F2006). Half volume of culture medium was exchanged every 3 days. U0126 (MCE, HY-12031) and Ceramide C6 (Santa cruz, sc-3527) were dissolved in DMSO and dissolved in medium without mitogens at a concentration of 10 μM.

### Immunohistochemistry

For fluorescence immunocytochemistry, NSPCs adhered to different precoated coverslips were fixed with 4% paraformaldehyde in 0.01 M phosphate-buffered saline (pH 7.4) for 2 hours at room temperature and blocked with 5% v/v fetal bovine serum or with 0.5% v/v Triton-X 100 (Sigma-Aldrich, ×100) in PBS. NSPCs were incubated with rabbit polyclonal to Ki67 (Abcam, 1:200), rabbit polyclonal to Doublecortin (Abcam, 1:200), rabbit polyclonal to glial fibrillary acidic protein (GFAP) (Proteintech Group, Inc, 1:100), rabbit polyclonal to Olig2 (Proteintech Group, Inc, 1:100), rabbit polyclonal to Sox2 (Abcam, 1:200), or mouse monoclonal to Nestin (Abcam, 1:250) overnight at 4 °C and then goat anti-rabbit Cy3 or anti-mouse FITC (Proteintech Group, Inc, 1:200), or donkey anti-rabbit FITC or anti-mouse Cy3 (Beyotime, 1:250) for 2 hours at room temperature. All cultures were counterstained with the DNA-binding dye 4′-6-Diamidino-2-phenylindole (DAPI, 2 mg/ml in PBS) for 10 minutes at room temperature. Then, Coverslips were mounted onto glass slides and the images were obtained from a Zeiss UV 780 Meta confocal microscope (Carl Zeiss), and examined by AxioVision 4 (Carl Zeiss AG) or Adobe Photoshop CS5 software.

### Western Blotting

NSPCs adhered to different precoated dishes were homogenized with lysis buffer containing 0.025 M Tris-HCl, pH 8.0, 0.15 M NaCl, 0.001 M EDTA, 1% Nonidet P-40, 10% glycerol; pH 7.4, and a protease inhibitor mixture (Complete, Roche). The protein concentration was measured by enhanced BCA Protein Assay Kit (Beyotime, P0010S). Proteins (15 μg/lane) were separated by 10% SDS-PAGE under reducing conditions and electroblotted to polyvinylidene difluoride membranes (Roche, 3010040001). Membranes were incubated in blocking with 5% skimmed milk or 3% bovine serum album (BSA) in TBST at room temperature for 2 hours. Membranes were then incubated with rabbit polyclonal to Doublecortin (Abcam, 1:2000), rabbit polyclonal to glial fibrillary acidic protein (GFAP) (Proteintech Group, Inc, 1:1000), rabbit polyclonal to Olig2 (Proteintech Group, Inc, 1:1000), mouse monoclonal to Nestin (Abcam, 1:2500), rabbit polyclonal to p44/42 MAPK(Erk1/2) (CST, 1:1000), rabbit polyclonal to Phospho-p44/42 MAPK (Erk1/2) (CST, 1:1000), or mouse monoclonal to GAPDH (Zsgb-bio, 1:1000) overnight at 4 °C. After incubation with peroxidase-conjugated (HRP)-conjugated secondary IgG (Zsgb-bio, 1:5000) for 2 hours at room temprature. All membranes were detected by ChemiDoc™ XRS+ imaging system using the Pierce Fast Western Blot Kits (Thermo Scientific, 35065). Densitometric measurement of each membrane was performed using Image Lab™ image acquisition and analyzed by Image Lab™ software. GAPDH, an internal control, was used to normalize the expression level of each protein.

### Cell Proliferation Assay

Cell proliferation was assessed by Cell Counting Kit-8, that determines the cell viability in cell proliferation through WST reduction assay (Dojindo, CK04-05), which detects dehydrogenase activities of viable cells. 100 μl of cell suspension (~10000 cells/well) was dispensed in a 96-well cell culture cluster with different pre-coated substrates, and they were incubated with 10% (v/v) WST solution for 2.5 h at 37 °C. Then, the absorbance of the culture medium at a test wavelength of 450 nm was determined using a microplate reader and a reference wavelength of 630 nm as well.

NSPCs adhered to different precoated dishes were dissociated using StemPro^®^ Accutase^®^ Cell Dissociation Reagent (Life Technologies, A1110501), and the dissociated cell suspensions were seeded in 24-well cell culture plates (2–5 × 10^5^ cells/ml) for fluorescence to visualise the percentage of Ki-67^+^ cells as described above.

### *In vitro* cytotoxicity assay

Lactate dehydrogenase (LDH) releasing levels were measured to evaluate cytotoxicity. LDH, released from injured cells in the medium, indicated the integrity of the cell membrane. Neurosphere culture supernatants were collected on day 3, 7 and 14. Cells were lysed with 2% Triton X-100 (Sigma, ×100) for 15 min to release all LDH from the cytoplasm after collecting the last supernatants. Cell lysis, served as positive controls, determined the maximal LDH release. LDH release was detected using the absorbance of the culture medium at a test wavelength of 450 nm with a microplate reader (Nanjing Jiancheng bioengineering institute, A020-2) according to the manufacturer’s instructions and it was shown as a rate of LDH released in the medium to total cellular (n = 4).

### Annexin-V Assay

Flow cytometry was used to detect Annexin-V staining via an Annexin V-FITC Apoptosis Detection Kit (Beyotime). Adherent cells dissociated using StemPro^®^ Accutase^®^ Cell Dissociation Reagent (Life Technologies, A1110501) were collected and processed in accordance with the manufacturer recommendation. After 10 minutes of incubation with Annexin V-FITC at room temperature, cells were resuspended and incubated in binding buffer. Then Propidium Iodide was added into cell suspension for 5 minutes on ice. Cells were analyzed using a FACScan flow cytometer (Becton Dickinson).

### Statistical methods

All data were showed as mean ± SEM and statistical analyses were carried out with SPSS v17.0 (SPSS Inc, Chicago, IL). Unpaired Student’s t test or one-way ANOVA followed by Tukey’s post hoc test was used to define statistical significance, and p < 0.05 was considered statistically significant.

## Additional Information

**How to cite this article**: Ge, H. *et al.* Poly-L-ornithine promotes preferred differentiation of neural stem/progenitor cells via ERK signalling pathway. *Sci. Rep.* **5**, 15535; doi: 10.1038/srep15535 (2015).
